# The Epstein-Barr Virus Lytic Protein BMLF1 Induces Upregulation of GRP78 Expression through ATF6 Activation

**DOI:** 10.3390/ijms22084024

**Published:** 2021-04-14

**Authors:** Lee-Wen Chen, Shie-Shan Wang, Chien-Hui Hung, Ya-Hui Hung, Chun-Liang Lin, Pey-Jium Chang

**Affiliations:** 1 Department of Respiratory Care, Chang-Gung University of Science and Technology, Chiayi 61363, Taiwan; lwchen@mail.cgust.edu.tw (L.-W.C.); flaghung@gmail.com (Y.-H.H.); 2 Department of Pediatric Surgery, Chang-Gung Memorial Hospital, Chiayi 61363, Taiwan; a483662@yahoo.com.tw; 3 School of Medicine, Chang-Gung University, Taoyuan 33302, Taiwan; 4 Graduate Institute of Clinical Medical Sciences, College of Medicine, Chang-Gung University, Taoyuan 33302, Taiwan; hungc01@mail.cgu.edu.tw; 5 Department of Nephrology, Chang-Gung Memorial Hospital, Chiayi 61363, Taiwan; linchunliang@cgmh.org.tw

**Keywords:** unfolded protein response, Epstein-Barr virus, lytic replication, BMLF1, GRP78, ATF6

## Abstract

The unfolded protein response (UPR) is an intracellular signaling pathway essential for alleviating the endoplasmic reticulum (ER) stress. To support the productive infection, many viruses are known to use different strategies to manipulate the UPR signaling network. However, it remains largely unclear whether the UPR signaling pathways are modulated in the lytic cycle of Epstein-Barr virus (EBV), a widely distributed human pathogen. Herein, we show that the expression of GRP78, a central UPR regulator, is up-regulated during the EBV lytic cycle. Our data further revealed that knockdown of GRP78 in EBV-infected cell lines did not substantially affect lytic gene expression; however, GRP78 knockdown in these cells markedly reduced the production of virus particles. Importantly, we identified that the early lytic protein BMLF1 is the key regulator critically contributing to the activation of the *grp78* gene promoter. Mechanistically, we found that BMLF1 can trigger the proteolytic cleavage and activation of the UPR senor ATF6, which then transcriptionally activates the *grp78* promoter through the ER stress response elements. Our findings therefore provide evidence for the connection between the EBV lytic cycle and the UPR, and implicate that the BMLF1-mediated ATF6 activation may play critical roles in EBV lytic replication.

## 1. Introduction

The unfolded protein response (UPR) is a major signaling pathway dealing with the endoplasmic reticulum (ER) stress that arises from excess accumulation of unfolded or misfolded proteins in the ER lumen [1,2,3]. In mammalian cells, activation of the UPR depends on three ER-resident transmembrane sensors including PKR-like ER kinase (PERK), inositol-requiring enzyme 1 (IRE1) and activating transcription factor 6 (ATF6). Normally, these senor proteins are inactive because their luminal domains are bound by a 78-kDa glucose-regulated protein (GRP78), also known as BiP or HSPA5. GRP78 is a chaperone protein belonging to the heat shock protein 70 kDa (HSP70) family, which acts as the master regulator of the UPR signaling pathway [4,5]. Under some pathophysiological conditions, the accumulation of unfolded or misfolded proteins may alter the binding preference of GRP78 in the ER lumen, consequently leading to the activation of PERK, IRE1 and ATF6 [1,2,3]. After dissociation from GRP78, activated PERK phosphorylates the eukaryotic translation initiation factor 2α (eIF2α), which attenuates global protein translation and thus alleviates the ER stress [6]. Conversely, the PERK-mediated eIF2α phosphorylation enables selective induction of translation of certain stress-mitigating proteins such as activating transcription factor 4 (ATF4) [6]. The second UPR sensor IRE1 is a unique enzyme that has both kinase and endoribonuclease activities. Upon activation, IRE1 triggers an unconventional splicing of the X-box binding protein 1 (XBP1) mRNA in the cytosol via its endoribonuclease activity [7,8]. Importantly, the gene product of the spliced XBP1 mRNA, sXBP1, is a functionally active transcription factor critically involved in upregulation of a variety of ER chaperones including GRP78. For the activation of the third UPR sensor ATF6, this membrane protein needs to be trafficked from ER to Golgi, where it undergoes the proteolytic cleavage and the subsequent release of its transcriptionally active N-terminal domain, ATF6-N [9,10]. The resultant ATF6-N then enters the nucleus to transactivate specific UPR target genes that encode ER chaperones and folding enzymes such as GRP78, GRP94 and protein disulfide isomerase (PDI). Although the UPR signaling pathways play important roles in cell survival and adaption via restoring ER homeostasis, the UPR can also induce cell apoptosis under excessive or prolonged stress of ER [3]. Since virus infection often disturbs ER homeostasis and leads to ER stress, different RNA or DNA viruses may have evolved distinct strategies to allow them to complete viral replication before the host cell is destroyed due to severe ER stress. Increasing evidence has demonstrated that several viruses utilize or differentially modulate UPR signaling pathways to support their latent or productive infections [11,12,13,14].

Epstein-Barr virus (EBV) is a double-stranded DNA virus that belongs to the γ-herpesvirus subfamily. Infection with this virus is closely associated with multiple human malignancies including Burkitt’s lymphoma [15], nasopharyngeal carcinoma [16], Hodgkin’s lymphoma [17], and gastric cancer [18], as well as other lymphoid and non-lymphoid cancers in immunocompromised hosts [19]. Like other herpesviruses, EBV displays two distinctive life cycles: latency and lytic replication [20,21]. Upon viral reactivation from latency, viral lytic genes are expressed in an orderly fashion: immediate-early (IE), early, and late gene expression, ultimately resulting in the assembly and egress of infectious virions. The BZLF1 and BRLF1 genes are two IE genes, which encode the transcriptional activators Zta (also known as ZEBRA or EB1) and Rta, respectively [22,23,24,25]. Numerous studies have shown that both Zta and Rta are the major latent-to-lytic switch proteins that trigger EBV reactivation by binding to and activating the viral lytic gene promoters [20,23,26,27]. In addition to Zta and Rta, the early lytic protein BMLF1 (also referred to EB2, SM or Mta), one of downstream targets of Rta, is also required for the viral gene expression and the viral lytic-cycle progression [28,29,30]. The BMLF1 protein is a multifunctional RNA-binding protein that may increase the viral or cellular gene expression through enhancing mRNA nuclear-cytoplasmic transport, mRNA splicing, mRNA stability or translation [28,31,32,33,34]. Following the viral DNA replication, both virion glycoproteins and capsids are considered to be highly expressed at late stages. Under the situation, it is possible that viral glycoproteins abundantly synthesized and accumulated in the ER could lead the host cell to ER stress-mediated death, thereby affecting the completion of the lytic cascade. To date, it is unclear whether ER stress occurs during the EBV lytic cycle, and how this virus maintains ER homeostasis to ensure a fully productive replication.

In this study, we aimed to explore the relationship between the EBV lytic cycle and the UPR signaling transduction. We found that the expression level of GRP78 was markedly upregulated during the viral lytic cycle, and the increased GRP78 expression was critically associated with the assembly or release of viral particles. Importantly, we identified that the viral lytic protein BMLF1 was the key regulator essential for the activation of the *grp78* gene promoter. Furthermore, we showed that the activation of the *grp78* promoter by BMLF1 was mainly mediated through ATF6 cleavage and activation. These results have led us to propose a new role of BMLF1 in modulating UPR signaling pathway during the EBV lytic cycle.

## 2. Results

### 2.1. The Expression of GRP78 Is Upregulated during the EBV Lytic Cycle

**Figure 1:**
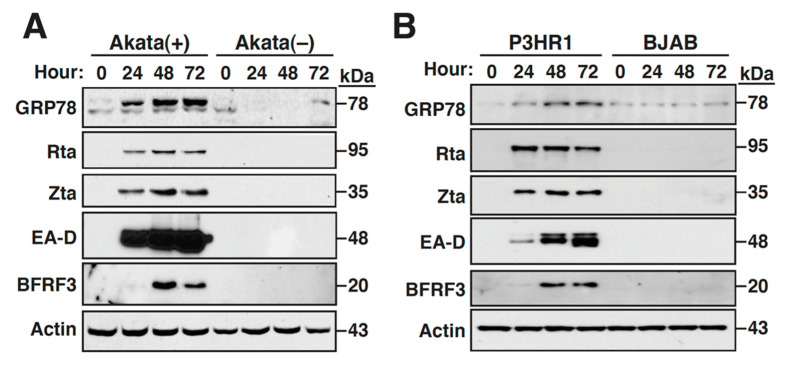
GRP78 expression is upregulated during the EBV lytic cycle. (**A**,**B**) All lymphoma cell lines, including Akata(+), Akata(−), P3HR1 and BJAB, were treated with SB plus TPA. At the indicated time points after treatment, the expressions of GRP78 and various EBV lytic proteins (including Rta, Zta, EA-D and BFRF3) in these treated cells were determined by Western blot analysis.

GRP78 is a major ER chaperon protein critically involved in protein folding and quality control of the ER [5]. Previously, we have shown that the GRP78 expression could be upregulated during the lytic cycle of Kaposi’s sarcoma-associated herpesvirus (KSHV), and found that the upregulation of GRP78 is essential for the KSHV lytic cycle [35]. To investigate whether the expression of GRP78 was also upregulated in the EBV lytic cycle, two latently EBV-infected lymphoma cell lines including Akata(+) and P3HR1 were treated with sodium butyrate (SB) plus 12-*O*-tetradecanoylphorbol-13-acetate (TPA) to induce the viral lytic replication. Additionally, two EBV-negative lymphoma cell lines, Akata(−) and BJAB, served as the control group in parallel experiments. As expected, the viral lytic proteins, including Rta, Zta, the early protein EA-D, and the late protein BFRF3, were detected only in Akata(+) and P3HR1 cells, but not in Akata(−) and BJAB cells, after treatment with SB plus TPA (Figure 1A,B). Under the SB/TPA-treated condition, we found that the expression level of GRP78 was consistently upregulated in Akata(+) and P3HR1 cells (Figure 1A,B), and the GRP78 upregulation was detected as early as 24 h after lytic induction. However, treatment with SB plus TPA did not significantly affect the expression levels of GRP78 in Akata(−) cells or in BJAB cells (Figure 1A,B). These results indicated that the GRP78 expression was induced during the EBV lytic cycle.

### 2.2. GRP78 Upregulation Is Important for Completion of the EBV Lytic Cycle

**Figure 2:**
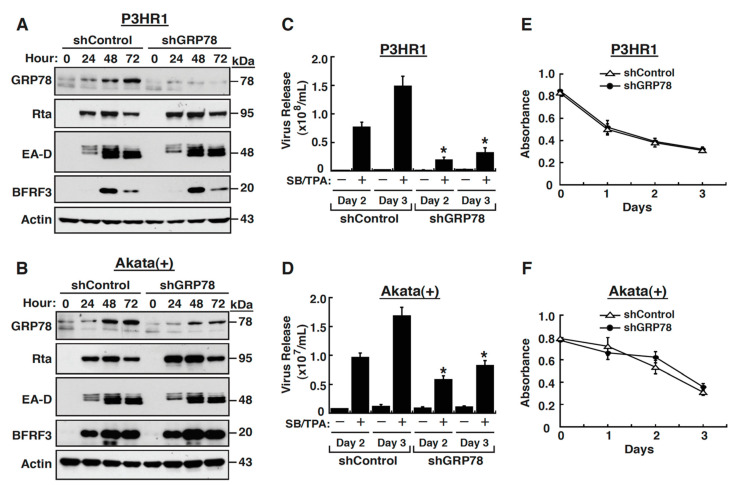
GRP78 upregulation is important for the EBV lytic cycle to go to completion. (**A**,**B**) Effect of GRP78 knockdown on the viral lytic protein expression in P3HR1 and Akata(+) cells. At the indicated time points after lentiviral infection and treatment with SB plus TPA, the expression levels of Rta, EA-D, BFRF3, and GRP78 in treated P3HR1 and Akata(+) cells were examined by Western blot analysis. shControl, control short hairpin RNAs; shGRP78, specific short hairpin RNAs against GRP78. (**C**,**D**) Effect of GRP78 knockdown on the release of virus particles from SB/TPA-treated P3HR1 cells and Akata(+) cells. The viral loads in culture supernatants on Day 2 and Day 3 after SB/TPA treatment were evaluated using quantitative PCR. * *p* < 0.05, for results compared to those of the shControl group at the same time points (*n* = 3). (**E**,**F**) Effects of GRP78 knockdown on the cell viability of P3HR1 and Akata(+) cells during the EBV lytic induction. Cell viability was analyzed by XTT assay (*n* = 3).

To determine the potential role of GRP78 upregulation in the EBV lytic cycle, knockdown of GRP78 was performed in P3HR1 and Akata(+) cells using the lentiviral vector expressing GRP78 shRNA (shGRP78). As compared to the cells with the control shRNA (shControl), P3HR1 or Akata(+) cells receiving shGRP78 exhibited a marked reduction in GRP78 levels (Figure 2A,B). At different time points (24, 48 and 72 h) after lentiviral infection and treatment with SB plus TPA, we found that GRP78 knockdown did not substantially affect the expression of the viral lytic proteins including Rta, EA-D and BFRF3 (Figure 2A,B). However, the virion production was significantly reduced in SB/TPA-treated P3HR1 and Akata(+) cells when the GRP78 expression was knocked down (Figure 2C,D), suggesting that GRP78 could play a crucial role in the assembly or release of EBV virions. The inhibition of virion production by GRP78 knockdown seemed to be more evident in P3HR1 cells than in Akata(+) cells (Figure 2C,D), which might be ascribed to more efficient GRP78 knockdown in P3HR1 cells than in Akata(+) cells (Figure 2A,B). Due to the fact that the GRP78 expression is often linked to cell survival, it might be possible that the reduced virion production in shGRP78-treated cells was related to increased cell death. To test this possibility, the viability of the shControl- and shGRP78-treated cells under lytic induction were examined by the cell proliferation (XTT) assay. In comparison with cells that received the control shRNAs, the cells receiving GRP78 shRNAs did not significantly affect their cell viability during the viral lytic cycle (Figure 2E,F). Taken together, these findings suggested that GRP78 upregulation may play an important role in the completion of the EBV lytic cycle.

### 2.3. Inhibition of the Viral DNA Synthesis and Late Gene Expression Has No Effect on the Sustained GRP78 Upregulation

**Figure 3:**
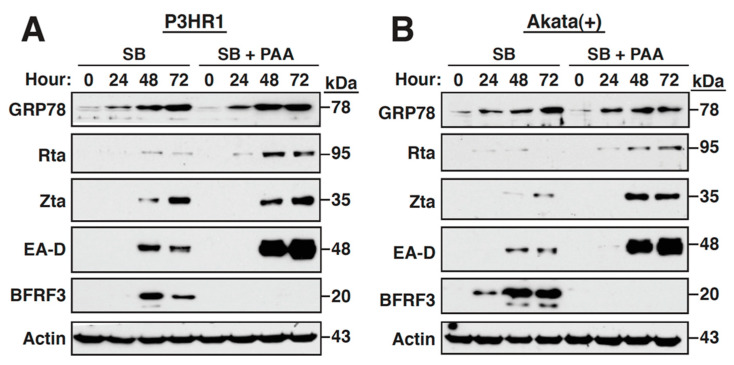
GRP78 upregulation occurs at the early stages of the EBV lytic cycle. P3HR1 cells (**A**) or Akata(+) cells (**B**) were treated with SB in the absence or presence of PAA (200 μg/mL). At different time points after treatment, cells were harvested, and the expressions of GRP78 and viral lytic proteins in these treated cells were analyzed by immunoblotting.

During the viral lytic cycle, a set of late proteins including various envelope glycoproteins need be abundantly synthesized in the ER. It could be possible that the abundant synthesis or accumulation of late proteins in the ER might be the potential stimulator for GRP78 upregulation. To explore the contribution of viral late proteins to GRP78 upregulation, P3HR1 cells or Akata(+) cells were treated with SB in combination with phosphonoacetic acid (PAA), a specific inhibitor of EBV DNA synthesis. As expected, PAA treatment completely abolished the expression of the viral late protein BFRF3 in P3HR1 or Akata(+) cells, but did not reduce the expression of the IE or early proteins such as Rta, Zta and EA-D (Figure 3A,B). Under the conditions, we found that PAA treatment did not substantially affect GRP78 upregulation in these two cell lines during the lytic induction. These results suggested that GRP78 upregulation is initiated before the viral DNA synthesis and late gene expression.

### 2.4. The Early Lytic Protein BMLF1 Sufficiently Activates the GRP78 Promoter in Lymphoma Cell Lines

**Figure 4:**
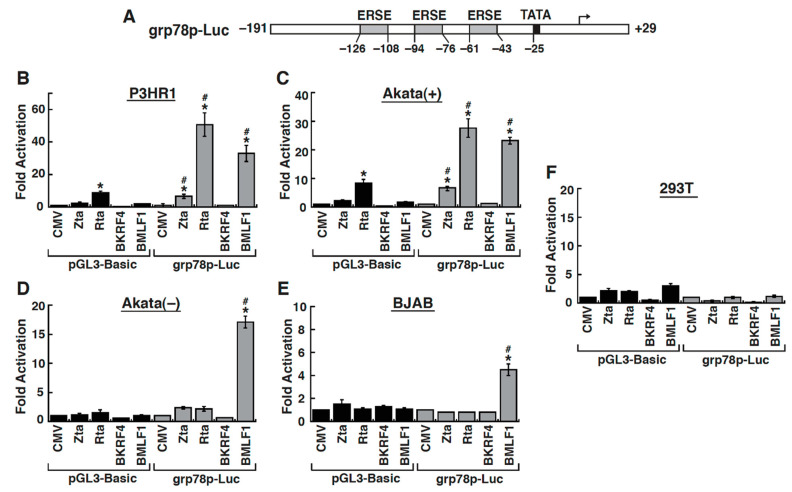
BMLF1 significantly activates the *grp78* gene promoter in lymphoma cell lines including P3HR1, Akata(+), Akata(−) and BJAB, but not in an epithelial cell line 293T. (**A**) Schematic diagram of the *grp78*p-Luc reporter construct. The *grp78*p-Luc reporter construct contains the *grp78* gene promoter region from −191 to +29, which encompasses three ERSE elements. (**B**–**F**) Effect of Zta, Rta, BKRF4 or BMLF1 on activation of the *grp78*p-Luc reporter construct in P3HR1, Akata(+), Akata(−), BJAB or 293T cells. Thirty hours after co-transfection, cells were harvested and measured for luciferase activity. The fold activation of the reporter construct was calculated as the luciferase activity in the presence of the individual expression plasmid divided by the luciferase activity in the presence of the empty vector control (CMV). All data are presented as mean ± SD from three independent experiments. *, *p* < 0.05, for results compared to those with the empty vector control; #, *p* < 0.05, for results compared to those with the control pGL3-Basic reporter.

To investigate whether specific viral lytic proteins were required for GRP78 upregulation, several possible candidates including Zta, Rta, BMLF1 and BKRF4 were tested for their ability to activate the *grp78* gene promoter. For these selective lytic proteins, Zta, Rta and BMLF1 have been previously reported to function as the transcriptional or post-transcriptional regulators of numerous viral or cellular genes [23,25,28]. Although BKRF4 protein is an ill-defined tegument protein [36], it might potentially contribute to some biological actions in the EBV lytic cycle. In the experiments, the luciferase reporter construct that harbors the *grp78* promoter region from −192 to +29, designated *grp78*p-Luc, was utilized in the transient co-transfection assay (Figure 4A). It is noteworthy to mention that there are three tandem copies of the ER stress response element (ERSE) located in the *grp78* promoter region of the reporter construct [10]. When the *grp78*p-Luc reporter construct or the control pGL3-Basic reporter construct was co-transfected with the expression plasmid encoding Zta, Rta, BMLF1 or BKRF4 into P3HR1 cells or Akata(+) cells, we found that overexpression of Zta or BMLF1 in cells could significantly activate the luciferase reporter activity of the *grp78*p-Luc reporter construct, but not pGL3-Basic (Figure 4B,C). Although Rta had a high background reporter activation (up to 8-fold) for the control pGL3-Basic reporter in P3HR1 or Akata(+) cells, we did observe that the activation of the *grp78*p-Luc reporter construct by Rta was much higher than that of pGL3-Basic (Figure 4B,C). These results indicated that Zta, Rta or BMLF1 was able to activate the *grp78* promoter in P3HR1 or Akata(+) cells.

Next, we determined whether ectopic expression of Zta, Rta or BMLF1 could sufficiently activate the *grp78* promoter in EBV-negative cells. Two lymphoma cell lines including Akata(−) and BJAB cells were then used in transient co-transfection and reporter assay. Our results revealed that the *grp78*p-Luc reporter construct was activated only by BMLF1, but not Zta or Rta, in Akata(−) cells (Figure 4D) or in BJAB cells (Figure 4E). These data implicated that BMLF1 could be the key factor essential for GRP78 upregulation in lymphoma cell lines. Surprisingly, we did not find the activation of the *grp78*p-luc reporter construct by BMLF1 in 293T cells, an EBV-negative epithelial cell line (Figure 4F). The failure to activate the *grp78* promoter by BMLF1 in 293T cells might be associated with lack of some specific downstream mediators in the cell line.

### 2.5. The ER Stress Response Element within the GRP78 Gene Promoter Is Essential for BMLF1-Mediated Activation

**Figure 5:**
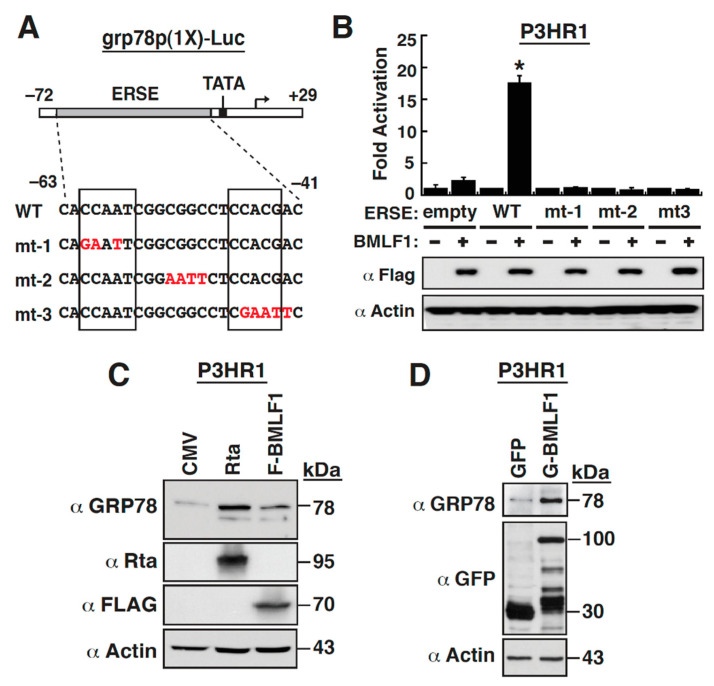
BMLF1 activates the *grp78* gene promoter through the ERSE element. (**A**) Schematic representation of the *grp78*p(1X)-Luc reporter construct. Note that only a single copy of the ERSE element in the *grp78*p(1X)-Luc reporter construct. Point mutations in the ERSE element of the reporter construct are indicated. (**B**) Transcriptional activation of the reporter constructs encompassing the wild-type or mutant ERSE element by BMLF1. The indicated reporter constructed were cotransfected with the empty vector control or the F-BMLF1 expression plasmid into P3HR1 cells for 30 h. Cells were harvested and measured for luciferase activity. Western blot analysis was performed to detect the F-BMLF1 expression in transfected cells using anti-FLAG antibody. *, *p* < 0.05, for results compared to those with the empty vector control (*n* = 3). (**C**) Effect of Rta or BMLF1 on the GRP78 expression in P3HR1 cells. The expression plasmids encoding Rta or F-BMLF1 were individually transfected into P3HR1 cells for 30 h. The expression levels of GRP78, Rta and F-BMLF1 in these transfected cells were evaluated by Western blot analysis. (**D**) Effect of GFP-BMLF1 on the GRP78 expression. P3HR1 cells were transfected with the expression plasmid encoding GFP or GFP-tagged BMLF1 (GFP-BMLF1). Thirty hours after transfection, the GFP-positive cells were sorted by flow cytometry and the sorted cells were used for Western blots to detect the GRP78 expression.

The ERSE elements in the *grp78* promoter are essential for the UPR transcriptional induction [10], and each ERSE contains a tripartite structure CCAAT-N9-CCACG, where N9 represents a 9-bp GC rich region located between two conserved sequence motifs [37,38]. To further clarify whether an intact ERSE in the *grp78* promoter was truly required for the BMLF1-mediated activation, the *grp78* promoter fragment from −72 to +29, which contains only an ERSE element, was cloned into pGL3-Basic, and the resultant reporter construct was designated as *grp78*p(1x)-Luc (Figure 5A). As compared to pGL3-Basic, the *grp78*p(1x)-Luc construct was still able to be activated by BMLF1 in P3HR1 cells (Figure 5B). Point mutations at the left CCAAT box (mt−1), the middle box (mt−2), or the right CCACG box (mt−3) in the conserved ERSE element of the *grp78*p(1x)-Luc construct were then created (Figure 5A). Transient-reporter analysis in P3HR1 cells revealed that all these ERSE mutants completely lost their responses to BMLF1 (Figure 5B), indicating that the BMLF1-mediated activation of the *grp78* promoter required an intact ERSE element.

We next examined whether overexpression of BMLF1 alone in P3HR1 cells sufficiently increased endogenous GRP78 expression (Figure 5C). Additionally, due to the reason that Rta is an upstream transcriptional activator of BMLF1, we also overexpressed Rta in P3HR1 cells to examine the expression levels of GRP78. Western blot analysis revealed that Rta or BMLF1 could significantly enhance GRP78 expression in P3HR1 cells (Figure 5C). Similarly, as compared to P3HR1 cells expressing green fluorescence protein (GFP), cells expressing GFP-tagged BMLF1 (GFP-BMLF1) also produced higher levels of GRP78 protein (Figure 5D).

### 2.6. Mapping of the BMLF1 Regions Responsible for the GRP78 Promoter Activation

**Figure 6:**
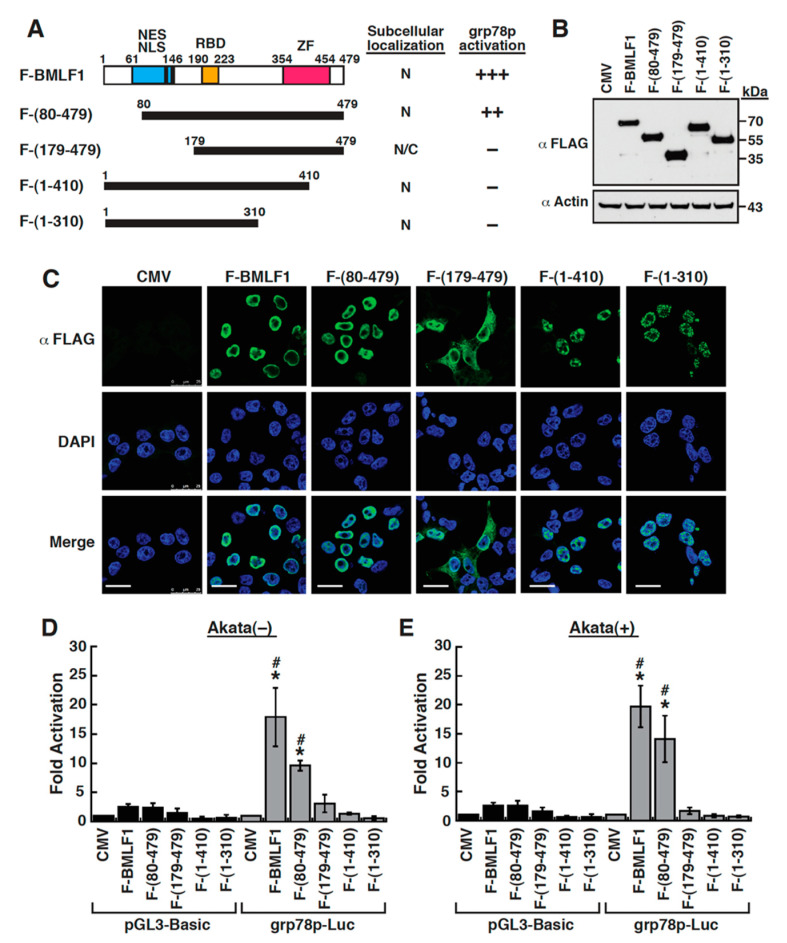
Both the N- and C-terminal portions of BMLF1 are critically required for the transcriptional activation of the *grp78* gene promoter. (**A**) Schematic diagram of BMLF1 and its deletion constructs. Specific functional motifs or domains of BMLF1 are indicated as follows: NES, nuclear export signal (aa 61–146); NLS, nuclear localization signal (aa 127–130 and aa 143–145); RBD, RNA-binding domain (aa 190–223); ZF, putative zinc-finger motif (aa 354–454). The subcellular localization of the wild-type and mutant BMLF1 proteins, and their ability to activate the *grp78* promoter are summarized in the diagram. N, nucleus; N/C, both nucleus and cytoplasm. The ability to activate the *grp78*p-Luc reporter construct is represented by “+”. (**B**) Western blots analysis of the wild-type and mutant BMLF1 constructs in 293T cells. The expression of β-actin was used as a loading control. (**C**) Confocal microscopic analysis of the wild-type and mutant BMLF1 constructs in 293T cells. 293T cells were transfected with the indicated plasmids encoding FLAG-tagged BMLF1 deletion constructs. Thirty hours after transfection, cells were immunostained with anti-FLAG antibody and analyzed by confocal microscopy. Scale bars, 20 μm. (**D**,**E**) Transcriptional activation of the *grp78*p-Luc reporter constructs by wild-type and mutant BMLF1 proteins in Akata(−) cells or in Akata(+) cells. In these experiments, the *grp78*p-Luc reporter construct were co-transfected with the expression plasmids encoding BMLF1 deletions into Akata(−) or Akata(+) cells. Cells were harvested 30 h after cotransfection and measured for luciferase activity. *, *p* < 0.05, for results compared to those with the empty vector control; #, *p* < 0.05, for results compared to those with the control pGL3-Basic reporter (*n* = 3).

BMLF1 is a 479-aa protein, which contains two nuclear localization signals (NLSs; aa 127–130 and aa 143–145), a nuclear export signal (NES) located within the N-terminal region between aa 61 and 146, and a RNA-binding domain (RBD) located at the middle region from aa 190 to 223 [31,39]. In addition to these functional domains or motifs, a zinc-finger domain could be predicted at the C-terminal domain of BMLF1 from aa 354 to 454 (Figure 6A). To further map the regions of BMLF1 essential for activating *grp78* promoter, a series of BMLF1 deletion mutants were constructed (Figure 6A) and their ability to activate the *grp78* promoter was examined. These BMLF1 deletion mutants include two N-terminal deletion mutants BMLF1-(80–479) and BMLF1-(179–479), and two C-terminal deletion mutants BMLF1-(1–410) and BMLF1-(1–310) (Figure 6A). The expression and the subcellular localization of these constructed BMLF1 proteins in 293T cells were determined by Western blot analysis (Figure 6B) and immunofluorescence analysis (Figure 6C), respectively. Among these deletion mutants, the BMLF1-(179–479) mutant that lacks the NLS motif was distributed in both the nucleus and the cytoplasm, whereas the other mutants were located in the nucleus (Figure 6C). It should be noted that although the C-terminal deletion mutants including BMLF1-(1–410) and BMLF1-(1–310) were localized in the nucleus, they formed punctate structures (Figure 6C). As shown in Figure 6D,E, the full-length BMLF1 could activate the *grp78* promoter by approximately 18-fold in Akata(−) cells and 20-fold in Akata(+) cells; however, the C-terminal deletion mutants, BMLF1-(1–410) and BMLF1-(1–310), completely lost their ability for the *grp78*p activation. For the N-terminal deletion mutants, the BMLF1-(80–479) mutant still retained 50 to 70% of the ability of the full-length BMLF1 to activate the *grp78* promoter in Akata(−) or Akata(+) cells (Figure 6D,E). However, further N-terminal deletion to the position 179, which removes the NLS and NES motifs, completely lost its ability to activate the *grp78* promoter (Figure 6D,E). Our results therefore suggested that the C-terminal zing-finger domain as well as the N-terminal NLS or/and NES motif were required for activating the *grp78* promoter.

### 2.7. The PERK- and IRE-1-Mediated UPR Signaling Pathways Are Not Activated during the EBV Lytic Cycle

**Figure 7:**
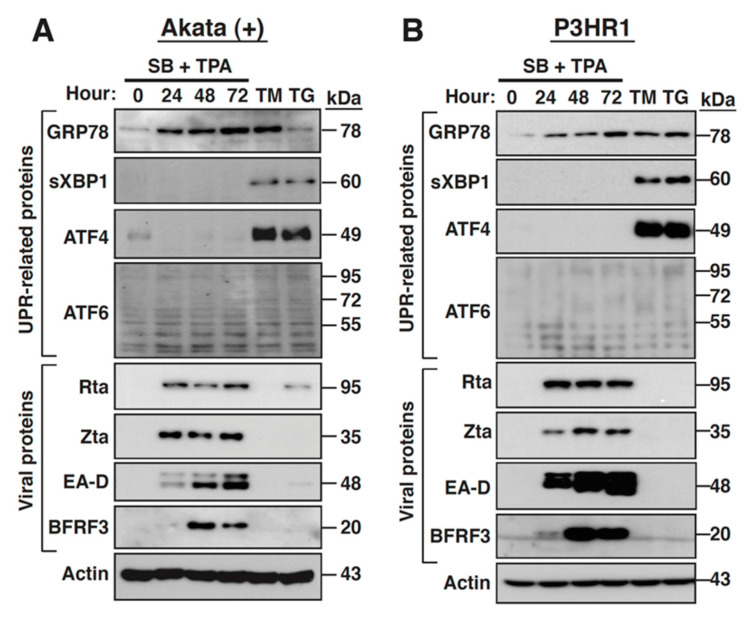
The PERK-ATF4 and IRE1-sXBP1 signaling pathways are not substantially activated during the EBV lytic cycle. The EBV lytic cycle in Akata(+) cells (**A**), or in P3HR1 cells (**B**) was induced by SB plus TPA. At the indicated time points after SB/TPA treatment, the protein expression of specific UPR effectors (including GRP78, sXBP1, ATF4 or ATF6) and viral lytic proteins (including Rta, Zta, EA-D and BFRF3) in treated cells were evaluated by Western blot analysis. Cells treated with tunicamycin (TM; 5 μg/mL) or thapsigargin (TG; 5 μM) for 24 h served as positive controls for UPR induction. Note that endogenous ATF6 in Akata(+) cells or in P3HR1 cells was difficult to be clearly detected using anti-ATF6 antibody.

The GRP78 upregulation promoted us to investigate whether specific UPR signaling pathway was activated during the EBV lytic cycle. We here examined the expressions of sXBP1 (a downstream effector of IRE-1), ATF4 (a downstream effector of PERK) and cleaved ATF6 (an active effector of ATF6 signaling) in SB/TPA-treated Akata(+) and P3HR1 cells. To ensure the induction of these UPR effectors, two well-known ER stress inducers, tunicamycin (TM) and thapsigargin (TG), were also used as positive stimuli to treat Akata(+) and P3HR1 cells (Figure 7A,B). Western blot analysis revealed that although GRP78 upregulation was consistently detected during the EBV lytic cycle, we did not observe the induction of sXBP1 and ATF4 expression in SB/TPA-treated Akata(+) or P3HR1 cells (Figure 7A,B). The failure to detect the expression of sXBP1 and ATF4 in SB/TPA-treated cells was unlikely to be the result of defective URP signaling in these cells because treatment with TM or TG markedly induced the expression of sXBP1 and ATF4 (Figure 7A,B). Noteworthily, we could not clearly detect endogenous ATF6 in Akata(+) or P3HR1 cells that were treated with SB/TPA, TM, or TG using the commercially available anti-ATF6 antibodies (Figure 7A,B and data not shown), suggesting that ATF6 could be expressed at low levels in these cells.

### 2.8. The Upregulation of GRP78 Expression by BMLF1 Is Mediated through ATF6 Activation

**Figure 8:**
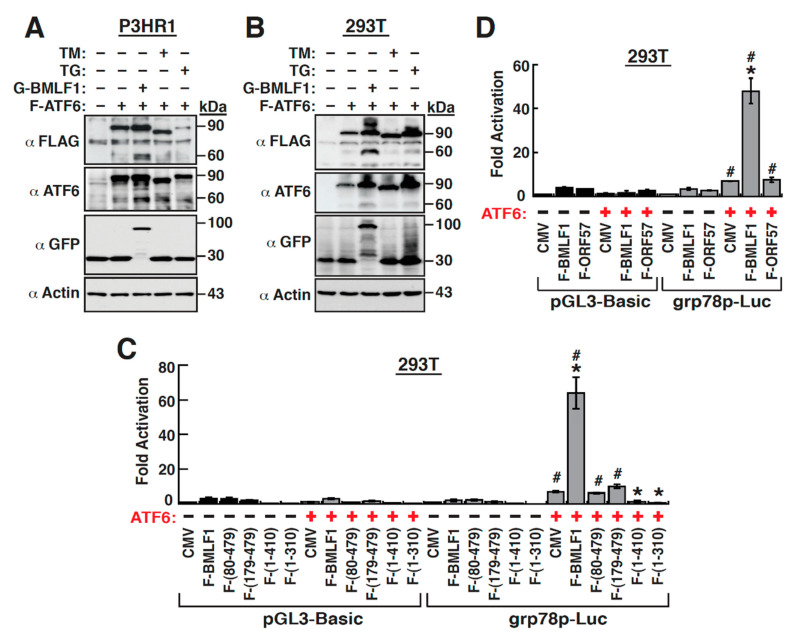
BMLF1 promotes the proteolytic cleavage and activation of the transfected ATF6. (**A**) Effect of GFP-BMLF1 on the proteolytic cleavage of transfected F-ATF6 in P3HR1 cells. Equal amounts of the F-ATF6 expression plasmid and the GFP- or GFP-BMLF1-expressing plasmid were co-transfected into P3HR1 cells. Thirty hours after co-transfection, cell lysates were prepared and then subjected to Western blot analysis using the indicated antibodies. Treatment of F-ATF6-transfected cells with tunicamycin (TM; 5 μg/mL) or thapsigargin (TG; 5 μM) for 24 h served as controls. (**B**) Effect of BMLF1 on the proteolytic cleavage of ATF6 in 293T cells. All experimental conditions were performed in the same way as mentioned above except 293T cells used here. (**C**) Transcriptional activation of the *grp78* gene promoter by various BMLF1 constructs in the presence or absence of F-ATF6 in 293T cells. The *grp78*p-Luc reporter construct or the pGL3-Basic reporter control was cotransfected with BMLF1 deletion constructs in the presence or absence of the F-ATF6 expression plasmid into 293T cells. A luciferase reporter assay was performed 30 h after cotransfection. *, *p* < 0.05, for results compared to those with the empty vector control (CMV); #, *p* < 0.05, for results compared to those of the group without F-ATF6 transfection (*n* = 3). (**D**) Effect of the KSHV ORF57, a homolog of the EBV BMLF1, on activation of the *grp78*p-Luc reporter construct. *, *p* < 0.05, for results compared to those with the empty vector control (CMV); #, *p* < 0.05, for results compared to those of the group without F-ATF6 transfection (*n* = 3).

Since the *grp78* gene is known as a downstream target of ATF6, we therefore sought to determine whether the BMLF1-mediated GRP78 upregulation was associated with ATF6 activation. To achieve this aim, P3HR1 cells were co-transfected with the plasmid encoding FLAG-tagged ATF6 (F-ATF6) and the GFP-BMLF1 expression plasmid or the empty vector control (Figure 8A). In parallel experiments, cells transfected with the F-ATF6 expression plasmid were treated with TM or TG, which served as the control groups (Figure 8A). Western blot analysis using either anti-FLAG or anti-ATF6 antibody revealed that GFP-BMLF1 substantially increased the proteolytic cleavage of F-ATF6, yielding 60-kDa cleaved products. (Figure 8A). The cleavage of F-ATF6 could be also mildly detected by treating the cells with TM or TG (Figure 8A). Particularly, similar results for the increased proteolytic cleavage of F-ATF6 by BMLF1 were also detected in 293T cells (Figure 8B). Due to the observation that the *grp78*p-Luc reporter construct could not be activated by BMLF1 in 293T cells (Figure 4F), it therefore raised the possibility that the level of endogenous ATF6 in 293T cells could be too low to support the BMLF1-mediated activation of the *grp78* promoter. To test this possibility, the *grp78*p-Luc reporter construct (200 ng) was cotransfected with the BMLF1 expression plasmid (200 ng), in combination with a relatively low amount (50 ng) of the F-ATF6 expression plasmid, into 293T cells. We found that cotransfection of the F-ATF6 expression plasmid significantly restored the activation of the *grp78* promoter by BMLF1 (Figure 8C). To further explore the involvement of ATF6 in the activation of *grp78* promoter by BMLF1, different BMLF1 mutants were included in transient-reporter assay (Figure 8C). Since ATF6 is an inducer of *grp78* promoter, overexpression of F-ATF6 in 293T cells could increase the *grp78* promoter activity by 11-fold. We here found that both the N-terminal deletion mutants including BMLF1-(80–479) and BMLF1-(179–479) could not further activate the *grp78* promoter in the presence of F-ATF6 (Figure 8C). Unexpectedly, coexpression of the C-terminal deletions including BMLF1-(1–410) and BMLF1-(1–310) contrarily attenuated the ATF6-mediated transactivation of the *grp78* promoter, suggesting that these two mutants act as dominant negative mutants under the conditions (Figure 8C). Additionally, we also examined the ability of the KSHV ORF57 protein, a homolog of the EBV BMLF1 [40], to activate the *grp78* promoter. Unlike BMLF1, the KSHV ORF57 did not enhance the *grp78*p activation in the presence of F-ATF6 in 293T cells (Figure 8D). Collectively, these data demonstrated that BMLF1 specifically activated the *grp78* promoter through ATF6 signaling pathway.

**Figure 9:**
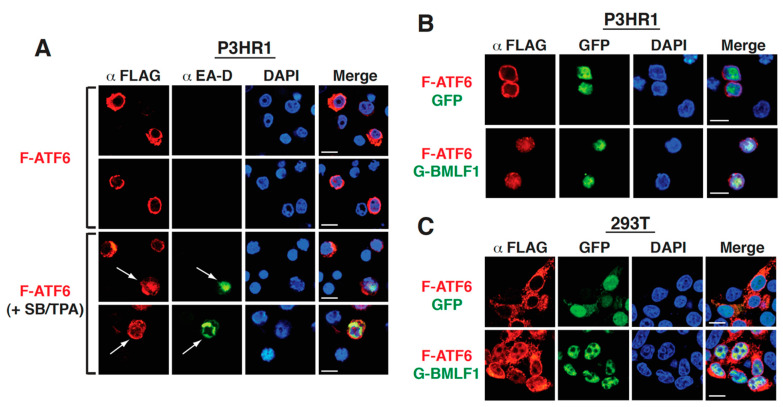
BMLF1 induces nuclear translocation of F-ATF6. (**A**) Representative confocal microscopic images of P3HR1 cells that were transfected with the F-ATF6 expression plasmid, and then untreated or treated with SB plus TPA. Forty hours after transfection and treatment, cells were fixed and dual labeled with an anti-FLAG antibody (red) and an anti-EA-D antibody (green). White arrows indicate the co-staining of F-ATF6 and EA-D in the same cell. Scale bars, 10 μm. (**B**) Confocal microscopic images of P3HR1 cells that were co-transfected with the F-ATF6 expression plasmid and the GFP or GFP-BMLF1 expression plasmid. Thirty hours after transfection, the distribution of F-ATF6 in the presence of GFP or GFP-BMLF1 in P3HR1 cells were examined by confocal microscopy. Scale bars, 10 μm. (**C**) Confocal microscopic images of 293T cells that were co-transfected with the F-ATF6 expression plasmid and the GFP or GFP-BMLF1 expression plasmid. The distribution of F-ATF6 in the presence of GFP or GFP-BMLF1 in 293T cells were examined 30 h after transfection. Scale bars, 10 μm.

Since the key step for ATF6 activation is the nuclear translocation of the cleaved ATF6-N, we monitored the subcellular localization of F-ATF6 using confocal fluorescence microscopy to further confirm our results. In P3HR1 cells, we observed that the transfected F-ATF6 protein was mainly localized in the cytoplasm under normal conditions (Figure 9A). However, after treatment with SB plus TPA, an enhanced nuclear translocation of F-ATF6 was specifically detected in cells expressing the lytic protein EA-D (Figure 9A). This finding supported that ATF6 activation occurred during viral reactivation. Furthermore, when the F-ATF6 expression plasmid was cotransfected with the GFP-BMLF1 expression plasmid into P3HR1 cells, a considerable amount of F-ATF6 was detected in nuclei of the co-transfected cells (Figure 9B). Similar results were also obtained in 293T cells showing that GFP-BMLF1, but not GFP, substantially enhanced the nuclear translocation of F-ATF6 (Figure 9C).

## 3. Discussion

In this report, we find that the expression of GRP78, a key UPR regulator, is significantly increased during the EBV lytic cycle, and the increased GRP78 could play an important role in the assembly or release of virus particles. We additionally identify that the early lytic protein BMLF1 is the key protein involved in the transcriptional activation of the *grp78* promoter. Mechanistic studies demonstrate that BMLF1 activates the *grp78* promoter mainly through promoting the cleavage and activation of ATF6. These findings may help to provide new insights into the reciprocal association between the ER homeostatic regulation and the EBV lytic cycle.

### 3.1. Expression and Function of GRP78 in the EBV Lytic Cycle

Due to the fact that all viral glycoproteins need to be synthesized, modified, folded and assembled in the ER, a large amount of viral proteins expressed or accumulated in the ER during virus infection may exceed the protein folding capacity of the ER. To ensure the proper folding and assembly of viral glycoproteins, different viruses may use distinct strategies to increase the expression of chaperones or folding enzymes in the ER [11,12,14]. GRP78 is the major chaperone and the UPR regulator in the ER, which also has the ability to maintain ER calcium homoeostasis. We here showed that the GRP78 expression is upregulated at the early stages of the EBV lytic cycle (Figure 1 and Figure 2), and inhibition of the viral DNA synthesis and late gene expression by PAA did not alter its sustained upregulation (Figure 3). Previous studies have also revealed that cells with other human herpesviruses, including KSHV, human cytomegalovirus (HCMV), or varicella-zoster virus (VZV), exhibited elevated GRP78 expression during the viral productive cycle [35,41,42,43]. In particular, the GRP78 upregulation also occurred at the early lytic stages of KSHV and HCMV. In the case of KSHV, two early viral proteins designated as ORF47/45-A and ORF47/45-B have been shown to activate GRP78 expression [35]. For HCMV, the IE protein IE1-72 is known to directly bind to and activate *grp78* promoter [44]. Herein, we identified that the viral early protein BMLF1 is involved in GRP78 upregulation during the EBV lytic cycle (Figure 4).

To explore the significance of GRP78 upregulation in the EBV lytic replication, we performed genetic knockdown of GRP78. Our results revealed that knockdown of GRP78 markedly reduced the virus particle production in EBV-infected cells that underwent lytic induction; however, the steady-state expressions of all tested viral lytic proteins, including the IE, early and late proteins, were not significantly affected by GRP78 knockdown (Figure 2). According to these results, we concluded that the increased GRP78 expression during the viral lytic replication could play a critical role in the assembly or release of virus particles. As for other herpesviruses, previous studies have also suggested that elevated GRP78 levels were critically involved in the assembly or release of HCMV and KSHV during the viral lytic induction [35,41,42]. Buchkovich et al. [41,42] reported that depletion or knockdown of GRP78 during HCMV infection strikingly reduced the production of infectious particles, but not obviously affected the expression of viral proteins. In our earlier studies, we also showed that although different KSHV-infected cell lines might display variable responses to GRP78 knockdown during the viral lytic replication, a marked reduction in the production of viral particles was the common feature [35]. Based on these findings obtained from previous studies and the present study, we think that GRP78 upregulation could be a common event present in the lytic replication of herpesviruses (e.g., HCMV, KSHV and EBV), and the increased GRP78 expression may exert similar or conserved functions potentially important for the completion of the lytic cycle.

### 3.2. Relationships between the Three UPR Branches and the EBV Lytic Cycle

**Figure 10:**
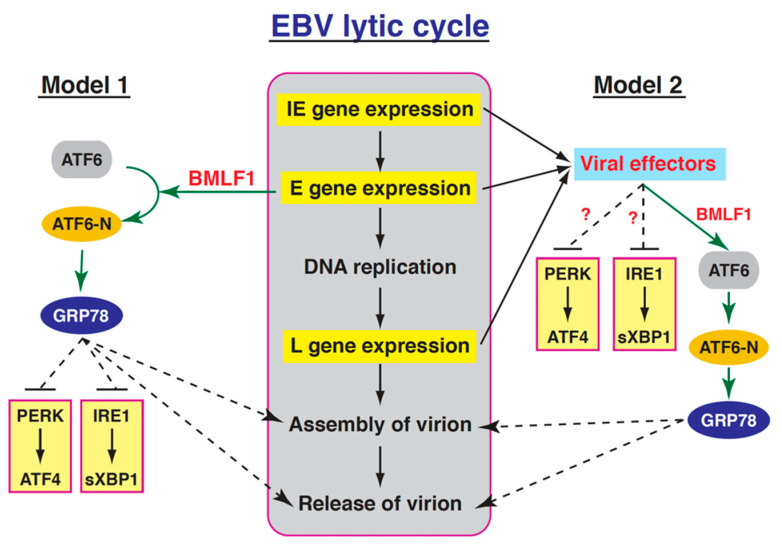
Proposed model for the regulation of the UPR signaling pathways in the EBV lytic cycle. There are two possibilities to explain activation of the ATF6-mediated pathway, but not the PERK- and IRE1-mediated pathways in the viral lytic cycle. In regard to the first possibility (Model 1), the BMLF1 protein is the only viral factor responsible for the regulation of UPR signaling cascades (e.g., ATF6 activation and GRP78 upregulation). The sustained high-level GRP78 expression may subsequently help to maintain PERK and IRE1 in their inactive states. As for the second possibility (Model 2), besides BMLF1, some as-yet-unidentified viral proteins may be required to modulate UPR signaling pathways (e.g., inhibition of the PERK- or IRE1-mediated signaling) to favor the viral lytic cycle. Solid black lines with arrows, well-known pathways; green lines with arrows, the findings of this study; dashed lines, possible pathways proposed.

Many viruses are known to use different mechanisms to modulate the three UPR branches, including the PERK-, IRE1- and ATF6-mediateing signaling pathways, to benefit their productive infection. Although members of the herpesvirus family may differ with their abilities to regulate the UPR signaling pathways, they may share some common features to favor their replication. Studies on KSHV have revealed that this virus could induce all three UPR sensor proteins during the lytic cycle, but elicits minimal downstream signaling events including accumulation of ATF4 and sXBP1 proteins as well as transcription of the UPR target genes [45]. Although the detailed mechanism of how the downstream UPR transcription is repressed during the KSHV lytic cycle remains unclear, they proposed that specific viral proteins might be responsible for the regulation of these UPR arms [45]. Similarly, the productive HCMV infection also triggers ER stress, but selectively induces PERK and IRE1 pathways, but not ATF6 pathway [13,46]. As compared to KSHV and HCMV, the association between the EBV lytic cycle and the UPR signaling pathway is poorly elucidated. Although previous studies have ever shown that overexpression of sXBP1, an UPR effector, could trigger viral lytic induction in latently EBV-infected cells [47], it is largely unclear whether the UPR signaling pathways were concomitantly activated during the EBV lytic cycle. Similar to KSHV [45], we found that the expression of ATF4 (the downstream effector of PERK) and sXBP1 (the downstream effector of IRE1) could not be induced during the EBV lytic cycle (Figure 7). However, unlike KSHV, the ATF6-mediated signaling pathway appeared to be activated during the lytic cycle of EBV (Figure 8 and Figure 9). To explain why only ATF6 pathway is activated in the EBV lytic cycle, at least two possibilities could be proposed (Figure 10). First, EBV *per se* could not induce ER stress and the subsequent UPR activation during its lytic cycle (Figure 10, Model 1). However, to ensure enough levels of chaperones or folding enzymes for optimal viral replication, the viral protein BMLF1 is endowed with the ability to activate the GRP78 expression through ATF6 activation. The sustained GRP78 upregulation in the viral lytic cycle may function not only for the assembly or release of virus particles, but also for inactivating the UPR sensor proteins PERK and IRE1 (Figure 10, Model 1). Second, ER stress could be induced upon viral reactivation (Figure 10, Model 2). However, to benefit the viral production, specific viral proteins expressed from IE, early or late stages may act cooperatively to inhibit the activation of PERK- and IRE1-mediated signaling cascades (Figure 10, Model 2). Concomitantly, the viral early protein BMLF1 is responsible for stimulating ATF6 activation, which causes upregulation of GRP78 expression (Figure 10, Model 2). In future research, more work is needed to distinguish these two models.

### 3.3. Activation of the GRP78 Promoter by BMLF1 through ATF6

BMLF1 is a multifunctional regulatory protein that may influence transcription, RNA stability, splicing, nuclear export and translation [28,31,32,33,34]. We here demonstrated that BMLF1 activated the *grp78* promoter in transient-reporter analysis (Figure 4). Although the KSHV ORF57 was reportedly to have similar activities to BMLF1 [40], we did not find the activation of *grp78* promoter by ORF57 protein (Figure 8D). These observations suggest that BMLF1 possesses a unique activity in modulation of specific gene expression. Since the BMLF1-mediated transactivation of *grp78* promoter did not occur in 293T cells (Figure 4F), the BMLF1 protein is unlikely to be a transcriptional activator directly binding to the *grp78* promoter DNA. In the study, we showed that activation of the *grp78* promoter by BMLF1 is mainly mediated through ATF6.

ATF6 is considered as a predominant factor in activating *grp78* promoter during the UPR activation. We here demonstrated that BMLF1 was able to promote the cleavage and activation of F-ATF6 in co-transfected P3HR1 and 293T cells (Figure 8A,B). Additionally, coexpression of F-ATF6 could restore the BMLF1-mediated transactivation of *grp78* promoter in 293T cells (Figure 8C). So far, the mechanism of how BMLF1 induces the cleavage and activation of ATF6 still remains to be determined. Since ATF6 activation requires at least two processes including the ER-to-Golgi translocation and the proteolytic cleavage by specific enzymes [9], it is possible that BMLF1 may directly or indirectly regulate these processes to activate ATF6.

When the profiles of activation of the *grp78* promoter by wild-type and mutant BMLF1 proteins in lymphoma cells and in 293T cells were compared, some differences could be observed between them. First, we failed to detect the activation of *grp78* promoter by BMLF1 in 293T cells under normal conditions (Figure 4F); however, ectopic expression of ATF6 rescued the BMLF1-mediated transactivation of the *grp78* promoter (Figure 8C). Accordingly, ATF6 would be a key mediator in the BMLF1-mediated *grp78*p activation and might be expressed at extremely low level in 293T cells under normal conditions. Second, we noticed that in 293T cells, the N-terminal deletion mutant BMLF1-(80–479) completely lose its ability to activate the *grp78* promoter, even in the presence of co-transfected F-ATF6 (Figure 8C). However, the BMLF1-(80–479) mutant still retained about 50–70% activity of the wild-type BMLF1 in lymphoma cells (Figure 6). It could be possible that in lymphoma cells, BMLF1 might have another way to regulate *grp78*p activation. Third, we found that the C-terminal deletion mutants including BMLF1-(1–410) and BMLF1-(1–310) displayed dominant negative effects on the ATF6-mediated *grp78*p activation in 293T cells (Figure 8C). In other words, overexpression of BMLF1-(1–410) or BMLF1-(1–310) inversely suppressed the ATF6-mediated activation of *grp78* promoter in 293T cells. It is possible that some factors essential for ATF6 activation could be sequestered by BMLF1-(1–410) or BMLF1-(1–310) in 293T cells, consequently blocking the ATF6-mediated activation of *grp78* promoter. Despite the fact that the wild-type and mutant BMLF1 proteins may have differential effects on the *grp78*p activation in lymphoma cells and in 293T cells, our results clearly demonstrate that ATF6 acts as an important mediator.

In summary, the present study provides evidence for the relationship between the UPR signaling and the EBV lytic cycle, and shows that the viral early protein BMLF1 activates GRP78 upregulation through activation of the UPR senor ATF6.

## 4. Materials and Methods

### 4.1. Cell Cultures and Chemical Reagents

P3HR1 and Akata(+) are two EBV-positive Burkitt’s lymphoma cell lines [48,49]. Akata(−) is an EBV-negative cell clone isolated from Akata(+) cells [50]. BJAB is an EBV-negative B-cell lymphoma cell line [51]. All lymphoma cell lines were cultured in RPM1 1640 (#11875085; Gibco, Thermo Fisher Scientific, Waltham, MA, USA) supplemented with 10% fetal bovine serum (FBS; #10437028; Gibco, Thermo Fisher Scientific, Waltham, MA, USA). HEK293T (293T), a human embryonic kidney cell line [52], was cultured in high-glucose Dulbecco modified Eagle medium (#11965084; Gibco, Thermo Fisher Scientific, Waltham, MA, USA) supplemented with 10% FBS. For viral lytic induction, cells were treated with 3 mM SB (#B5887; Sigma-Aldrich, St. Louis, MO, USA) alone or 3 mM SB plus 30 ng/mL TPA (#P8139; Sigma-Aldrich, St. Louis, MO, USA). The concentration of phosphonoacetic acid (#284270; Sigma-Aldrich, St. Louis, MO, USA) used in the study was 200 μg/mL. Tunicamycin (#T7765; Sigma-Aldrich, St. Louis, MO, USA) and thapsigargin (#T9033; Sigma-Aldrich, St. Louis, MO, USA) were typically used at 5 μg/mL and 5 μM, respectively, for 24 h.

### 4.2. DNA Transfection

All lymphoma cell lines were transfected by electroporation using the Neon Transfection System device (catalog no. MPK5000; Thermo Fisher Scientific, Waltham, MA, USA) and the Neon Transfection System 100 μL kit (catalog no. MPK10096; Thermo Fisher Scientific, Waltham, MA, USA). Briefly, 4 × 10^6^ cells, along with 10 or 20 μg of plasmid DNA, were resuspended in 100 μL of Buffer B (supplied in the Neon Transfection System 100 μL kit). After electroporation (1400 V, 20 ms, 2 pulses), the transfected cells were transferred into the culture medium (2 mL) in 6-well plates. Transient DNA transfection in 293T cells was performed using Lipofectamine 2000 according to the manufacturer’s instruction (catalog no. 11668019; Thermo Fisher Scientific, Waltham, MA, USA).

### 4.3. Plasmid Construction

Plasmids, including pCMV-Z, pRTS-Rta and *grp78*p-Luc were described previously [35,53]. The pCMV-FLAG-BMLF1, pCMV-FLAG-BKRF4 and pCMV-FLAG-ORF57 plasmids that encode BMLF1, BKRF4 and ORF57, respectively, with a N-terminal FLAG tag were generated by inserting the EBV BMLF1, the EBV BKRF4 or the KSHV ORF57 coding region into pFLAG-CMV-2 (#E7398; Sigma-Aldrich, St. Louis, MO, USA). To construct the expression plasmids encoding BMLF1 deletions, the indicated BMLF1 coding regions (Figure 6A) were cloned into pFLAG-CMV-2. The expression plasmid encoding GFP-BMLF1 was constructed by inserting the full-length BMLF1 coding regions into pEGP-C2 (#6083-1; Clontech Laboratories, Inc., Palo Alto, CA, USA). To generate the *grp78*p(1X)-luc reporter plasmid, the human *grp78* promoter element from positions −72 to +29 were inserted into pGL3-Basic (#E1751; Promega, Madison, WI, USA) at XhoI and HindIII sites. Point mutations in the *grp78* promoter of the *grp78*p(1X)-luc reporter plasmid were created using the QuickChange site-directed mutagenesis kit (#200524; Agilent Technologies, Palo Alto, CA, USA). The F-ATF6 expression plasmid was constructed by inserting full-length ATF6 cDNA into pFLAG-CMV-2.

### 4.4. Western Blot Analysis

Immunoblotting analysis was performed as mentioned previously [54]. Antibodies to Zta (sc-53904; Santa Cruz, Dallas, TX, USA), Rta (REF11-008; Argene, Verniolle, France), EA-D (sc-58121; Santa Cruz, Dallas, TX, USA), FLAG (A8592; Sigma-Aldrich, St. Louis, MO, USA), GRP78 (sc-13968; Santa Cruz, Dallas, TX, USA), GFP (G1544; Sigma-Aldrich, St. Louis, MO, USA), sXBP-1 (619502; BioLegend, San Diego, CA, USA), ATF4 (#11815; Cell Signaling, Boston, MA, USA), ATF6 (MAB6762; Abnova, Taipei, Taiwan)), and actin (sc-47778; Santa Cruz, Dallas, TX, USA) were purchased commercially. The rabbit anti-BFRF3 antibody was kindly provided by Dr. Shih-Tung Liu [55].

### 4.5. Lentivirus-Based Knockdown

All lentivirus-based vectors were obtained from the National RNAi Core Facility Platform at the Institute of Molecular Biology/Genomic Research Center, Academia Sinica (Taiwan). Preparation of lentiviral particles has been described previously [35]. Briefly, the lentiviral vector encoding *grp78* shRNA or control shRNA was cotransfected with pCMV-ΔR8.91 and pMD.G into 293T cells. After 24 h, the culture medium was changed with fresh medium. The lentivirus-containing media were collected on Day 2 and Day 3 after transfection. The target sequence of *grp78* shRNA is 5′-AGATTCAGCAACTGGTTAAAG. Lentivirus transduction into P3HR1 cells and Akata(+) cells was performed in the presence of Polybrene (#TR-1003; Sigma-Aldrich, St. Louis, MO, USA) at a final concentration of 8 μg/mL.

### 4.6. Quantitative Determination of Viral Particles

Quantification of encapsidated viral DNA was performed using the PCR-based TaqMan method as described previously [35]. Briefly, the culture media of the P3HR1 and Akata(+) cells were collected at Day 2 and Day 3 after treatment with SB plus TPA. To eliminate possible DNA contamination, the cell culture supernatants were first treated with DNase I (30 units/mL) for 1 h. DNase I (#M0303S) was obtained from New England Biolabs (Ipswich, MA, USA). After DNase treatment, the supernatants were further treated with 0.1% SDS (#L3771; Sigma-Aldrich, St. Louis, MO, USA) and 100 μg/mL proteinase K (#P8107S; New England Biolabs, Ipswich, MA, USA) at 37 °C to destroy virions, and viral DNA was extracted by the phenol-chloroform (1:1) method. The amount of the EBV DNA was measured by quantitative PCR using the BioRad iCycler iQ real-time PCR detection system (BioRad Laboratories, Hercules, CA, USA). The nucleotide sequences of the TaqMan probe (specific to the EBNA1 gene) and primers used in the study were 5′-FAM-AGGGAGACACATCTGGACCAGAAGGC-TAM (where FAN is 6-carboxyfluorescein and TAM is 6-carboxytetramethylrhodamine), 5′-TACAGGACCTGGAAATGGCC (forward primer), and 5′-TCTTTGAGGTCCACTGCCG (reverse primer).

### 4.7. Cell Viability Assay

The cell viability was evaluated using the XTT Cell Proliferation kit (#11465015001, Roche, Penzberg, Germany) according to the manufacturer’s instruction. The absorbance of the converted dye was measured at a wavelength of 450 nm using a microtiter plate reader. A reference wavelength of 630 nm was used to determine nonspecific readings. Experiments were carried out in duplicate and repeated three times.

### 4.8. Luciferase Assays

The reporter assays were done according to the manufacturer’s protocol for the luciferase reporter assay system (#E1501; Promega, Madison, WI, USA). The P3HR1, Akata or 293T cells were transfected with a fixed amount of plasmid DNA that included the reporter plasmid and the expression plasmids encoding effectors. The reporter assays were routinely performed 30 h after DNA transfection into P3HR1, Akata or 293T cells. Fold activation was calculated as the luciferase activity in the presence of effector divided by that in the absence of effector. All data shown in the study were obtained from at least three independent experiments.

### 4.9. Confocal Immunofluorescence Analysis

Confocal immunofluorescence analysis was performed as mentioned previously [35]. After P3HR1 cells or 293T cells were transfected with the expression plasmids encoding F-ATF6 or other proteins for 30 to 40 h, the transfected cells were fixed with 4% paraformaldehyde in phosphate-buffered saline (PBS) for 10 min and then washed with PBS three times for 5 min each time. The fixed cells were permeabilized with 0.1% Triton X-100 in PBS for 10 min. After the blocking step, the cells were incubated with anti-FLAG (1:200) or/and anti-EA-D (1:200) antibodies at 4 °C overnight. Following three further washing steps, appropriate secondary antibodies were added. DAPI (4′,6′diamidino-2-phenylindole) was used to label nuclear DNA of cells. Finally, the slides were mounted, and cells were examined with a Leica confocal laser scanning system (TCS-SP5II). Image processing was carried out using LAS AF Lite software (Leica, Wetzlar, Germany).

### 4.10. Statistical Analysis

All data were represented as mean values with standard deviations. Statistical analysis was performed using SPSS (Statistical Package for the Social Sciences) software version 18.0. Two-tailed Student’s t test was used to evaluate the significance of difference between groups. *p* < 0.05 was considered statistically significant.

## 5. Conclusions

In the present report, we find that a major UPR regulator GRP78 is markedly up-regulated during the viral lytic cycle, and demonstrate that the GRP78 upregulation could play a key role in the assembly or egress of virus particles. Furthermore, we show that the viral BMLF1 protein can induce GRP78 upregulation through activation of an UPR senor protein ATF6. Our study therefore proposes that ATF6 activation may have a beneficial impact on the viral propagation.

## Data Availability

All data presented in the study are available upon request from the corresponding author.

## Abbreviations

EBV	Epstein-Barr virus
BMLF1	BamHI-M leftward reading frame 1
ATF6	activating transcription factor 6
GRP78	78-kDa glucose-regulated protein
UPR	unfolded protein response
ER	Endoplasmic reticulum
PERK	PKR-like ER kinase
IRES	inositol-requiring enzyme 1
BiP	binding immunoglobulin protein
HSPA5	heat shock protein family A member 5
HSP70	heat shock protein 70 kDa
eIF2a	eukaryotic translation initiation factor 2α
ATF4	activating transcription factor 4
XBP1	X-box binding protein 1
GRP94	94-kDa glucose-regulated protein
PDI	protein disulfide isomerase
KSHV	Kaposi’s sarcoma-associated herpesvirus
SB	sodium butyrate
TPA	12-*O*-tetradecanoylphorbol-13-acetate
Zta	Z transcriptional transactivator
Rta	R transcriptional transactivator
PAA	phosphonoacetic acid
ERSE	ER stress response element
GFP	green fluorescence protein
TM	tunicamycin
TG	thapsigargin
HCMV	human cytomegalovirus
VZV	varicella-zoster virus
